# Unifying and versatile features of flavin-dependent monooxygenases: Diverse catalysis by a common C4a-(hydro)peroxyflavin

**DOI:** 10.1016/j.jbc.2023.105413

**Published:** 2023-11-02

**Authors:** Aisaraphon Phintha, Pimchai Chaiyen

**Affiliations:** School of Biomolecular Science and Engineering, Vidyasirimedhi Institute of Science and Technology (VISTEC), Wangchan Valley, Rayong, Thailand

**Keywords:** flavin, flavoenzyme, monooxygenase, C4a-hydroperoxyflavin, C4a-peroxyflavin, enzyme catalysis, enzyme mechanism, enzyme structure

## Abstract

Flavin-dependent monooxygenases (FDMOs) are known for their remarkable versatility and for their crucial roles in various biological processes and applications. Extensive research has been conducted to explore the structural and functional relationships of FDMOs. The majority of reported FDMOs utilize C4a-(hydro)peroxyflavin as a reactive intermediate to incorporate an oxygen atom into a wide range of compounds. This review discusses and analyzes recent advancements in our understanding of the structural and mechanistic features governing the enzyme functions. State-of-the-art discoveries related to common and distinct structural properties governing the catalytic versatility of the C4a-(hydro)peroxyflavin intermediate in selected FDMOs are discussed. Specifically, mechanisms of hydroxylation, dehalogenation, halogenation, and light-emitting reactions by FDMOs are highlighted. We also provide new analysis based on the structural and mechanistic features of these enzymes to gain insights into how the same intermediate can be harnessed to perform a wide variety of reactions. Challenging questions to obtain further breakthroughs in the understanding of FDMOs are also proposed.

Flavin-dependent enzymes utilize flavin derivatives—mostly FMN or flavin adenine dinucleotide (FAD)—which are derived from vitamin B2, as cofactors or substrates. These flavin cofactors exhibit remarkable versatility, as they can undergo one- or two-electron transfers, allowing the enzymes to adopt multiple redox states (1, 2, 3). The ability of flavins to exist in a transient radical state (*i.e.* flavin semiquinone) either through a natural redox cycle (reduced by a substrate) or light activation enables a wide range of challenging reactivities such as oxygen activation, C-C-bond formation, C-C-bond cleavage, and C-N bond formation (4, 5, 6, 7, 8). Flavin-dependent enzymes play pivotal roles in diverse catalytic reactions and biological processes involved in mostly redox metabolisms such as xenobiotic detoxification, biosynthetic pathways in plants and biosynthesis of microbial secondary metabolites, neural development in humans, and light-emitting reactions in bacteria. Non-redox reactions such as galactofuranose synthesis in bacteria and fungi can also be catalyzed by flavin-dependent enzymes (1, 2, 9, 10, 11, 12). Flavin-dependent enzymes, especially the flavin-dependent monooxygenases (FDMOs), have drawn significant attention in the fields of biochemistry and biotechnology due to their involvement in a broad spectrum of catalytic reactions. FDMOs function by incorporating a single atom of molecular oxygen into their substrates. These enzymes have great potential for use in various applications including the biosynthesis of valuable chemicals, as well as in drug metabolisms, biodetoxification, bioremediation, and biosensors (3, 13, 14).

FDMOs comprise a diverse group of enzymes that exhibit a wide variety of structures and functions. Based on the protein components, these enzymes can be divided into two main types: single-component and two-component monooxygenases. Single-component monooxygenases bind flavin constitutively; flavin reduction by NAD or NAD(P)H and substrate monooxygenation occur at the same active site. In contrast, two-component monooxygenases require a flavin reductase to generate a reduced flavin (15, 16). The reduced flavin is then transferred (can occur *via* diffusion) from a flavin reductase to the monooxygenase for subsequent oxygen activation and substrate monooxygenation (17, 18). FDMOs can be categorized into eight groups (groups A-H) based on their structural characteristics and functional properties (16, 19). They activate oxygen and generate reactive intermediates through flavin oxygenation reactions, forming either C4a-(hydro)peroxyflavin or flavin N5-peroxide as oxygenating reagents (Fig. 1) (7, 20, 21). Among the known FDMOs, most of the enzymes form C4a-(hydro)peroxyflavin, which plays a critical role in FDMOs as an oxygen donator *via* nucleophilic or electrophilic reactions. Herein, we focus on the reactions of FDMOs that generate C4a-(hydro)peroxyflavin as an intermediate. This intermediate allows FDMOs to catalyze a wide range of reactions including hydroxylation, Baeyer–Villiger oxidation, sulfoxidation, epoxidation, denitration, dehalogenation, halogenation reactions, and light emission (1, 16, 22). Understanding how different FDMOs control the remarkable diversity of C4a-(hydro)peroxyflavin would allow these enzymes to be engineered for a variety of impactful applications.

**Figure 1:**
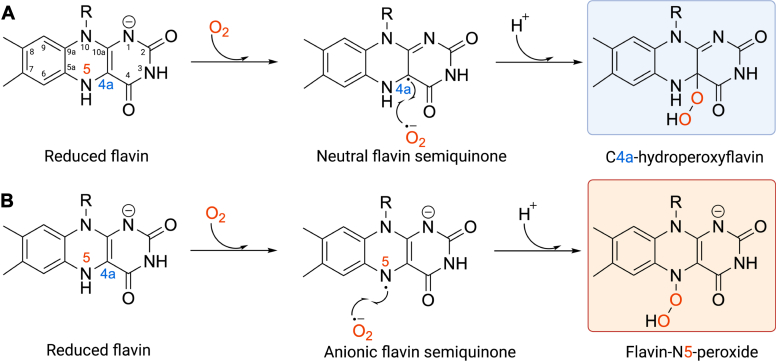
**Schematic representation of reactive flavin intermediates formation in FDMOs.***A*, C4a-hydroperoxyflavin formation. *B*, flavin-N5-peroxide formation. In both types of FDMOs and N5-adduct forming FDMOs, the enzyme-bound reduced flavin reacts with oxygen, resulting in the formation of superoxide and a flavin semiquinone radical pair. In the majority of FDMOs, this radical pair occurs at the C4a position, while in N5-adduct forming FDMOs, it occurs at the N5 position of the isoalloxazine ring. Subsequently, these radical pairs collapse to form either C4a-hydroperoxyflavin or flavin-N5-peroxide, depending on the specific type of FDMO. FDMO, flavin-dependent monooxygenase.

This review thus highlights the current state-of-knowledge of the reaction mechanisms and structures of extensively investigated FDMOs. The structural and functional properties, particularly common or unique features governing the versatility of the C4a-(hydro)peroxyflavin, are identified and used for explaining how the same common flavin intermediate can be used to manifest diverse reactions such as aromatic hydroxylation, N-hydroxylation, dehalogenation, halogenation, and light emission. A deep understanding of how FDMOs fine-tune their reactivities should be valuable in the future for designing FDMOs through enzyme engineering and creating potential biocatalysts for various applications.

## Unifying features of flavin-dependent monooxygenases

### Various protein foldings for recognizing flavins

**Figure 2:**
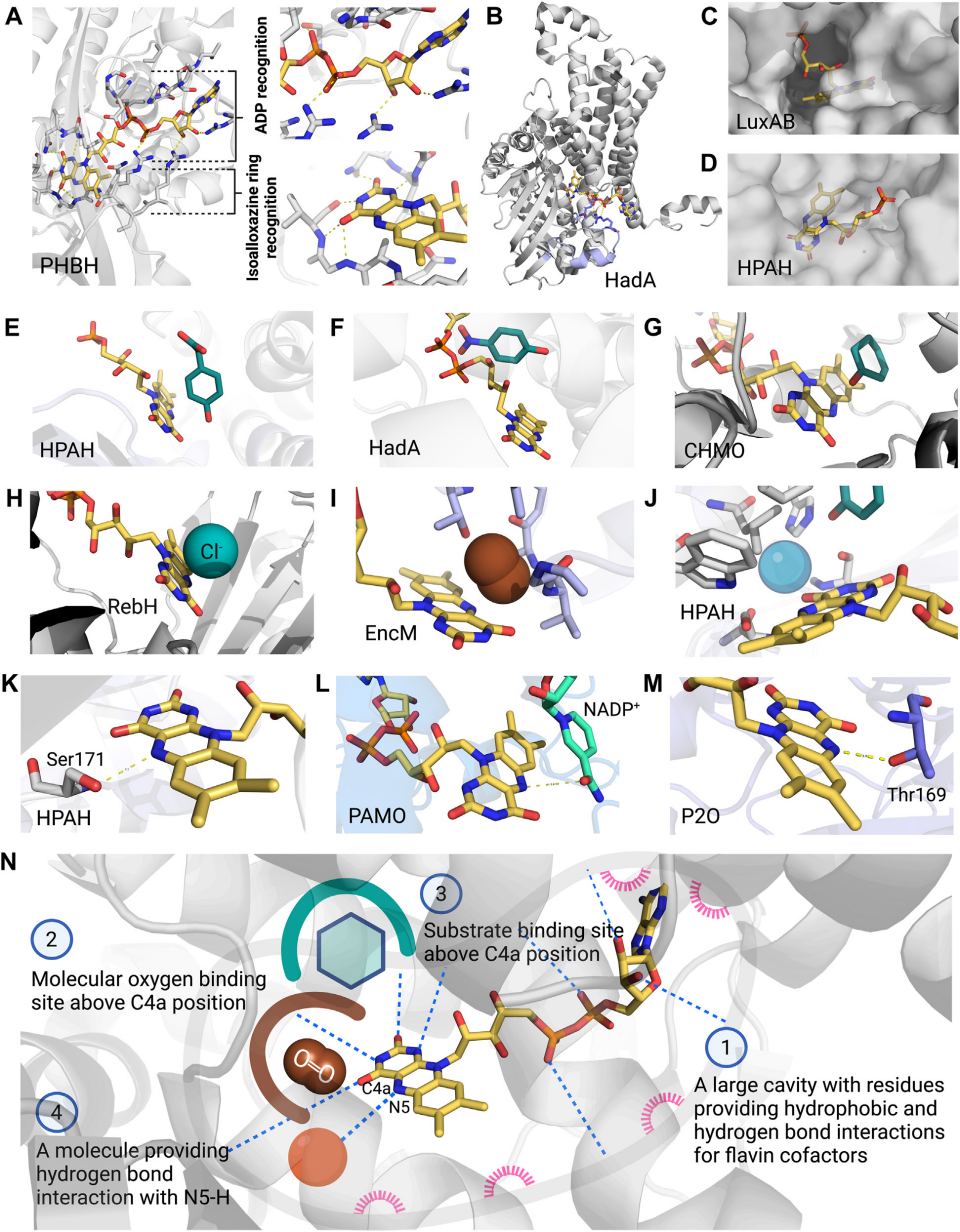
**Common features of FDMOs.***A*, FAD bound at the flavin-binding site of PHBH (PDB: 1PBE). *B*, flavin-binding loop (*purple*) in the HadA structure (PDB: 7E8P). *C*, FMN bound in the LuxAB structure (PDB: 3FGC). *D*, FMN bound in HPAH (PDB: 2JBT). *E*, substrate (*green sticks*)-binding site of HPAH (PDB: 2JBT). *F*, substrate-binding site of HadA (PDB: 7E8P). *G*, substrate-binding site of CHMO (PDB: 3UCL). *H*, substrate-binding site of tryptophan halogenase (RebH) (PDB: 2OA1). *I*, molecular oxygen bound (*brown spheres*) in the flavin-binding site of flavin N5-oxide stabilizing enterocin biosynthesis monooxygenase (PDB: 6FOQ) (Note that flavin N5-oxide stabilizing enterocin biosynthesis monooxygenase does not share common structural and flavin intermediate features as the other FDMOs in this panel. It is displayed here to provide reference for the oxygen-binding site). *J*, the predicted oxygen-binding site (*blue sphere*) in the HPAH structure (PDB: 2JBT). *K*, hydrogen bonding between N5-H and Ser171 in the HPAH structure (PDB: 2JBT). *L*, hydrogen bonding between N5-H and NADP^+^ in the PAMO structure (PDB: 2YLR). *M*, hydrogen bonding between N5-H and Thr169 in the pyranose-2-oxidase structure (PDB: 1TT0). *N*, the summary of key common features of FDMOs. CHMO, cyclohexanone monooxygenase; FAD, flavin adenine dinucleotide; FDMO, flavin-dependent monooxygenase; FMN, flavin mononucleotide; P20, pyranose 2-oxidase; PAMO, phenylacetone monooxygenase.

FDMOs comprise a diverse group of enzymes with a wide variety of structures and functions. The overall folding of FDMOs can be categorized into three different folding types including a Rossmann fold, TIM-barrel fold, and acyl-CoA dehydrogenase fold (16, 20). Despite the variations in their structures, FDMOs share a common trait—the utilization of a flavin cofactor for oxygen activation. These foldings provide structural arrangements that facilitate the recognition of flavin binding. FDMOs can utilize either FAD or FMN; their specificity is predominantly determined by two factors (1): spatial accommodation or the availability of space to accommodate the flavin cofactor and (2) binding site environment or the interactions within the binding site that enable the recognition of the ADP portion of the flavin molecule. Notably, this recognition pattern is a distinctive characteristic of enzymes with a Rossmann fold such as 3-hydroxy-benzoate 4-hydroxylase (PHBH) from *Pseudomonas fluorescens* and flavin-dependent halogenase (Thal) from *Streptomyces albogriseolus* (Fig. 2*A*) (16, 23, 24, 25).

The shared features among FDMOs recognizing ADP include interactions between polar and/or charged residues and the pyrophosphate and/or ribose sugar moieties of ADP (Fig. 2*A*). This interaction is particularly prominent in enzymes having the Rossmann fold mentioned above. Interestingly, for FDMOs which possess the acyl-CoA dehydrogenase fold instead of the Rossmann fold for recognizing FAD—such as group D: 4-hydroxyphenylacetate (HPA) 3-monooxygenase from *Escherichia coli* (HpaB) (26) and flavin-dependent dehalogenase (HadA) from *Ralstonia pickettii* DTP0602 (26)—these enzymes also possess similar interaction features as those with the Rossman fold within a segment designated as the “flavin binding loop” (Fig. 2*B*). This loop aids in the recognition of ADP, enabling these enzymes to bind specifically to FAD or FADH^-^.

Conversely, enzymes having the TIM-barrel fold (*e.g.*, groups C and H of FDMOs) contain binding sites where the isoalloxazine ring binds deeply within the protein structure such as bacterial luciferase (LuxAB) (Fig. 2*C*). Because this binding configuration lacks the necessary space to accommodate ADP binding, these FDMOs only bind to FMNH^-^ as their substrate.

An intriguing exception is observed in some cases of group D enzymes such as 3-HPA 4-hydroxylase from *Acinetobacter baumannii* (HPAH, the oxygenase component of HPAH or C2) and 4-HPA 3-monooxygenase (*Tt*HpaB) from *Thermus thermophilus* HB8, in which a pocket for the isoalloxazine ring is situated near the protein surface (Fig. 2*D*) (15). This structural arrangement allows the side chain of flavin cofactors to extend outward from the enzyme structure, enabling both FADH^-^ and FMNH^-^ to be utilized as substrates.

### Isoalloxazine binding site

The flavin-binding site within FDMOs typically comprises of a spacious pocket capable of accommodating a flavin cofactor. This pocket exhibits a combination of hydrophobic and polar regions (Fig. 2*A*). The hydrophobic characteristics of this pocket create an optimal environment for accommodating the three aromatic rings of the isoalloxazine ring. Simultaneously, the polar regions are finely tuned to interact with specific moieties of the isoalloxazine ring around the N1, N3, C2-carbonyl oxygen, and C4-carbonyl oxygen (Fig. 2*A*). These distinctive environments are organized by the side chains and/or amino acid residue backbones.

### Substrate-binding site orientation

The substrate-binding domain within FDMOs exhibits remarkable diversity, enabling FDMOs to proficiently catalyze a wide spectrum of reactions (16, 20). Despite the differences in the overall protein folding and variations in amino acid sequence, FDMOs maintain a consistent arrangement of substrate and flavin binding, positioning the substrate above the oxygen reacting site around the C4a position of the isoalloxazine ring on the *re*-side. This area is proposed to be the site where the reaction of reduced flavin and oxygen takes place, thus facilitating the generation of the C4a-(hydro)peroxyflavin intermediate (Fig. 2, *E*–*H*) (explained more in detail in the next section) (1, 7). This mode of substrate binding is commonly observed in all FDMOs in which the substrate oxygenation occurs *via* a terminal oxygen atom transfer from C4a-(hydro)peroxyflavin onto a substrate. This binding arrangement appears across a range of substrates with diverse properties, including size, polarity, and steric characteristics. Examples include binding sites of halide ions in tryptophan halogenases, aromatic substrates in hydroxylases and dehalogenases, as well as cyclohexyl ketone in cyclohexanone monooxygenase (CHMO) (Fig. 2, *E*–*H*) (27, 28, 29, 30, 31). This binding arrangement likely facilitates robust interactions between substrate and C4a-(hydro)peroxyflavin, thus enabling convenient oxygen atom transfer (7).

It should be noted that the mode of substrate binding at the *re*-side in C4a-(hydro)peroxyflavin-forming enzymes is not observed in FDMOs that form flavin N5-oxide. In the case of FDMOs that form flavin N5-oxide, the substrate was found at the *si*-face of the flavin, while the molecular oxygen is proposed to approach at the *re*-side (32). This was explained by results from computational calculations which demonstrated that the mechanism of substrate oxygenation by flavin N5-oxide is different from C4a-(hydro)peroxyflavin in which flavin N5-oxide at the *re*-side is brought closer to the substrate at the *si*-face *via* N5-inversion (32).

With greater availability of structural and mechanistic information regarding FDMOs, it would be interesting to conduct comparative investigations across a broader range of FDMOs with diverse reactions and substrate utilizations. The analysis may provide a deeper understanding of the controlling features of the enzyme and lead to the ability to modify specific properties of FDMOs.

### Oxygen activation

As described above, FDMOs exhibit a unique structural arrangement, with the isoalloxazine ring of flavin positioned deep into the structure (1, 16). A fundamental question arises: how does the buried flavin cofactor that is secluded from the solvent environment react with oxygen to form the reactive C4a-(hydro)peroxyflavin intermediate? Extensive investigations into the reaction mechanism of reduced flavin with molecular oxygen have shed light on this process. It has been proposed that molecular oxygen diffuses through multiple tunnels, reaching the oxygen-binding pocket located at the *re*-side of the isoalloxazine ring, near the C4a position (1, 7). This unique arrangement allows efficient formation of the C4a-(hydro)peroxyflavin intermediate, shields the intermediate from the surrounding solvent environment, and facilitates its prompt oxygen transfer reaction with the substrate (1, 16). Recent crystal structures of FDMOs including a stabilizing flavin N5-oxide monooxygenase involved in enterocin biosynthesis (flavin N5-oxide stabilizing enterocin biosynthesis monooxygenase) from *Streptomyces maritimus* (33) and a pyrimidine monooxygenase (RutA) from *E. coli* (32) have identified the oxygen-binding location at the *re*-side of the flavin (Fig. 2*I*). The data are also valuable in confirming the oxygen reaction site for other FDMOs such as HPAH and HadA (Fig. 2*J*) in which their oxygen reaction site have been identified by computational calculations (30, 31, 34). These data significantly advance our understanding of the oxygen activation reaction by FDMOs and demonstrate how these enzymes effectively control the reaction of reduced flavin and oxygen in order to generate the critical C4a-(hydro)peroxyflavin intermediate essential for substrate functionalization.

### Stabilization of C4a-(hydro)peroxyflavin

A distinctive feature of FDMOs is their ability to generate C4a-(hydro)peroxyflavin as an intermediate during their catalytic reactions. The stability of C4a-(hydro)peroxyflavin varies among the different enzymes. In most cases, this intermediate can only be detected using rapid kinetic studies (35, 36). The ability of FDMOs to stabilize C4a-(hydro)peroxyflavin is among the key distinctions of FDMOs from flavoenzyme oxidases in which the intermediate could not be detected during their catalytic cycles, either because it is not formed or because it rapidly decays (37, 38, 39). The exceptions were found for the reactions of pyranose 2-oxidase from *Trametes multicolor*, where the C4a-hydroperoxyflavin was detected during the oxidative half-reaction, and choline oxidase in which the flavin C4a-adduct could be detected by single-crystal spectroscopic method and protein crystallization (40, 41).

Comparison of the structures of FDMOs and oxidases is instrumental for identifying the critical factors within the C4a-N5 locus that stabilize the C4a-(hydro)peroxyflavin intermediate. Site-directed mutagenesis of HPAH demonstrated the significance of Ser171, located in close proximity to, and forming an H-bond with the N5, for stabilization of the C4a-hydroperoxyflavin intermediate (Fig. 2*K*). Replacing this residue with Ala resulted in a dramatic decrease in intermediate stabilization (42). Similar interactions were also found in the Thr residues of *Tt*HpaB (43), chlorophenol 4-monooxygenase (TftD) from *Burkholderia cepacia* AC1100 (44), and HadA (31). As these enzymes are those which can stabilize C4a-(hydro)peroxyflavin well during their catalytic cycles (7, 45), the H-bonding interaction around flavin N5 is thus thought to be important for C4a-(hydro)peroxyflavin stabilization. Interestingly, in the case of Baeyer–Villiger monooxygenases (BVMOs) and N-hydroxylating monooxygenases (NMOs), despite the absence of an amino acid residue capable of forming a hydrogen bond with the N5, the binding of NADP^+^ is required for stabilization of C4a-(hydro)peroxyflavin (for periods of up to hours, that is, in the reactions of CHMO from *Acinetobacter* sp. NCIMB 9871 (46) and NMO from *Aspergillus fumigatus* (SidA) (47)). As the co-complex structure of phenylacetone monooxygenase—one of the BVMOs—and NADP^+^ shows the hydrogen bonding between an amide oxygen of NADP^+^ and N5 hydrogen, this H-bonding around the N5 position is also important for C4a-(hydro)peroxyflavin stabilization in this case (Fig. 2*L*) (48, 49, 50, 51). Additionally, the hydrogen bonding of N5 with Thr also exists in pyranose 2-oxidase, facilitating the stabilization of the C4a-hydroperoxyflavin (Fig. 2*M*) (40, 52).

The interactions around the flavin N5 position are different in different enzyme systems. In the case of bacterial luciferase, it was previously suggested that hydrogen bonding around the N5 position might form with a carbonyl oxygen of the *cis*-peptide linkage between α-Ala74 and α-Ala75 (53). Based on our analysis of the structures of flavin-dependent halogenases, no amino acid residues capable of forming a hydrogen bond with the N5 hydrogen was identified. Instead, the crystal structures of various flavin-dependent halogenases, such as tryptophan 7-halogenase (PDB: 2AQJ), tryptophan 5-halogenase (PDB: 2WET), tryptophan 6-halogenase (PDB: 7CU1), pyrrole halogenase (PDB: 5BVA), phenolic halogenase (PDB: 3E1T), and aliphatic halogenase (PDB: 3I3L) show the presence of water molecules in close proximity with the N5. These water molecules are proposed to participate in the formation of a water network and engage in hydrogen bonding interactions with the N5 hydrogen, as suggested by molecular dynamics simulations of Thal (24). Conversely, this stabilizing environment is not found in the structure of glucose oxidase, in which the C4a-(hydro)peroxyflavin could not be detected using rapid-kinetics (38, 54). Altogether, the presence of a group capable of forming a hydrogen bond with N5 hydrogen appears to be crucial for C4a-(hydro)peroxyflavin stabilization and possibly is a distinguishing feature between FDMOs and flavoenzymes capable of stabilizing the intermediate from classic flavoenzyme oxidases which cannot stabilize it.

In summary, FDMOs display remarkable diversity in structure and function. Nevertheless, they share four common features including (1) flavin cofactor recognition features, (2) well-defined environment around the isoalloxazine ring to accommodate reactions with molecular oxygen and substrate binding, (3) molecular interactions around the C4-N5 locus, such as the presence of residues, ligand, or a small molecule capable of forming hydrogen bonds with the N5 hydrogen of the flavin (Fig. 2*N*). These essential features enable FDMOs to effectively generate the C4a-(hydro)peroxyflavin intermediate, which plays a vital role in substrate functionalization.

## The reaction versatility of C4a-(hydro)peroxyflavin in various FDMOs

The C4a-(hydro)peroxyflavin intermediate plays a critical role in the reactions of FDMOs and its diversified reactivities can be attributed to the different nucleophilic and electrophilic characteristics of the -OH transfer in different enzymes. The ability of FDMOs to control the reactivity of this intermediate gives rise to a wide range of reactions including hydroxylation, Baeyer–Villiger oxidation, sulfoxidation, epoxidation, denitration, dehalogenation, halogenation, and light-emission reactions (1, 16, 22). Here, we highlight how selected FDMOs direct the same chemical species to catalyze different reactions.

### Aromatic hydroxylation

**Figure 3:**
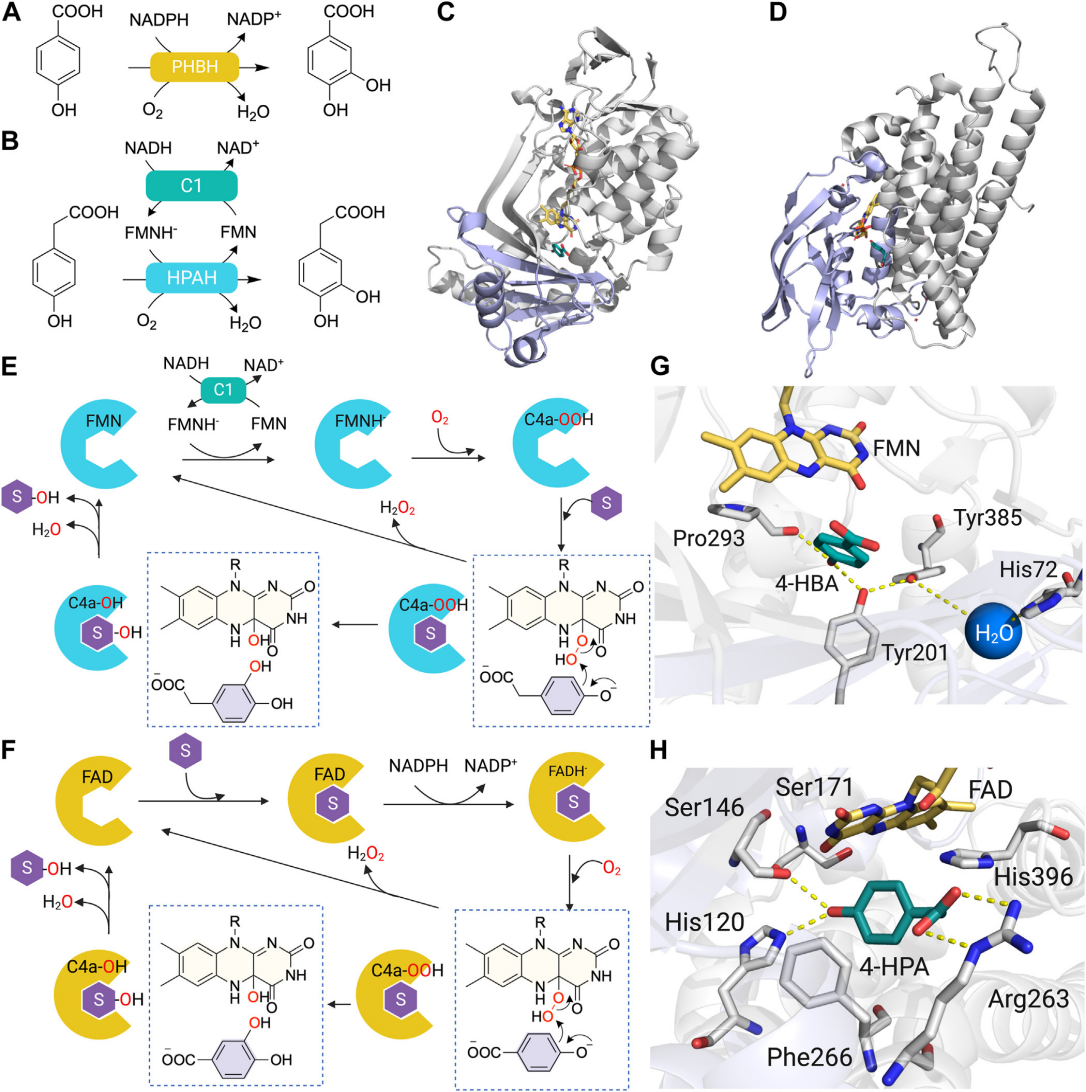
**Structures and reaction mechanisms of FDMOs catalyzing hydroxylation.***A*, the reaction of PHBH catalyzing the hydroxylation of 4-hydroxybenzoate (4-HBA). *B*, the reaction of HPAH catalyzing the hydroxylation of 4-hydroxyphenylacetate (4-HPA). *C*, the overall structure of PHBH with FAD and 4-hydroxybenzoate bound (PDB: 1PBE) consisting of a flavin-binding domain (*gray*) and a substrate-binding domain (*purple*). *D*, the overall structure of HPAH with FMN and *p*-hydroxyphenylacetate bound (PDB: 2JBT) consists of a flavin-binding domain (*gray*) and a substrate-binding domain (*purple*). *E*, the overall catalytic reaction of HPAH. *F*, the overall catalytic reaction of PHBH. *G*, key residues in the flavin- and substrate-binding sites of PHBH. *H*, key residues in the flavin- and substrate-binding sites of HPAH. FAD, flavin adenine dinucleotide; FDMO, flavin-dependent monooxygenase; FMN, flavin mononucleotide.

Numerous FDMOs, both single-component and two-component systems, have been documented for their ability to catalyze aromatic hydroxylation (15, 16, 20, 55, 56). To reveal the key characteristics that empower FDMOs to perform their catalytic hydroxylation, this section focuses on the extensively studied FDMOs which catalyze aromatic hydroxylation including PHBH (23) to represent the single-component FDMOs (group A FDMOs) and HPAH (57) to represent the two-component FDMOs (group D FDMOs). For their specific reactions, PHBH catalyzes the conversion of 4-hydroxybenzoate to form 3,4-dihydroxybenzoate, while HPAH catalyzes the conversion of 4-HPA to form 3,4-dihydroxyphenylacetate (Fig. 3, *A* and *B*).

The overall structure of PHBH and HPAH primarily consists of the flavin-binding domain and the substrate-binding domain (Fig. 3, *C* and *D*) (16, 20, 30, 55, 58). PHBH and HPAH possess several distinctive features. For instance, PHBH does not require a flavin reductase, whereas HPAH does (Fig. 3, *A* and *B*) (57, 59). In the PHBH reaction, flavin reduction and substrate oxygenation takes place within the same active site. The presence of an aromatic substrate can enhance the rate of the flavin reaction in PHBH, while for HPAH, this event takes place at the flavin reductase component (Fig. 3, *E* and *F*) (60). The binding of an aromatic substrate can induce conformational changes in PHBH, facilitated by the hydrogen bond networks of His72, Tyr201, Pro293, Tyr385, and water molecules (Fig. 3*G*) (58). These conformational changes promote the movement of the isoalloxazine ring to a position where NADPH can reduce the bound FAD, a state known as the "out" conformation (61). Further details regarding the conformational changes in PHBH can be found in the previously published references. (15, 55, 61, 62).

Although PHBH and HPAH generate the same reactive C4a-hydroperoxyflavin (C4a-OOH) intermediate as previously mentioned, the mechanisms to facilitate the subsequent electrophilic aromatic substitution reaction with the aromatic substrate are different. In PHBH, the hydrogen bonding networks facilitate the flavin conformational changes in PHBH as previously mentioned, lowering the p*K*a of a *p*-hydroxyl group of 4-hydroxybenzoate by approximately 2 units and positioning the C3 carbon of the substrate towards the C4a position of the isoalloxazine ring (Fig. 3*F*) (58, 60). These arrangements enable electrophilic aromatic substitution by PHBH, leading to the production of 3,4-dihydroxybenzoate.

In HPAH, pH-dependence studies and measurement of hydroxylation rate constants using transient kinetics indicate that the hydroxylation rate constants are constant around pH 6 to 10 (36). Additionally, ^19^F NMR data illustrates a noticeable decrease in the p*K*a value for the 4-HPA substrate when it is bound compared to its free form (63). These observations indicate that the bound aromatic substrate possibly exists in the deprotonated state throughout this pH range. The substrate deprotonation which directly supports the electrophilic aromatic substitution by C4a-OOH is facilitated by the surrounding residues within the substrate-binding site, such as His120 and Ser146 which are crucial for proper substrate orientation (Fig. 3*H*) (15, 64). The 4-HPA hydroxylation activity of HPAH is completely eliminated when His120 is substituted with a neutral or negatively charged amino acid. In contrast, the hydroxylation activity is maintained when positively charged amino acids replace His120 (64). This highlights the importance of a positive charge at position 120 for stabilizing the phenolate form of the substrate, thus facilitating an efficient aromatic hydroxylation. In addition, when the Ser146 residue is mutated to Ala, the rate of 4-HPA hydroxylation is significantly reduced (64). These findings highlight the importance of His120 and Ser146 to the enzyme activity (64). Following the hydroxylation, C4a-hydroxyflavin (C4a-OH) is formed and subsequently eliminates water to result in the oxidized flavin. As HPAH does not bind to oxidized flavin well, the enzyme resets to the apoenzyme form, ready for the next catalytic cycle (Fig. 3*E*).

Overall, the active site architecture plays a vital role in controlling the hydroxylase activity of both PHBH and HPAH. It governs proper substrate-binding geometry poising it for phenolic group deprotonation which facilitates the -OH transfer from C4a-OOH *via* electrophilic aromatic substitution mechanisms.

### N-hydroxylation

**Figure 4:**
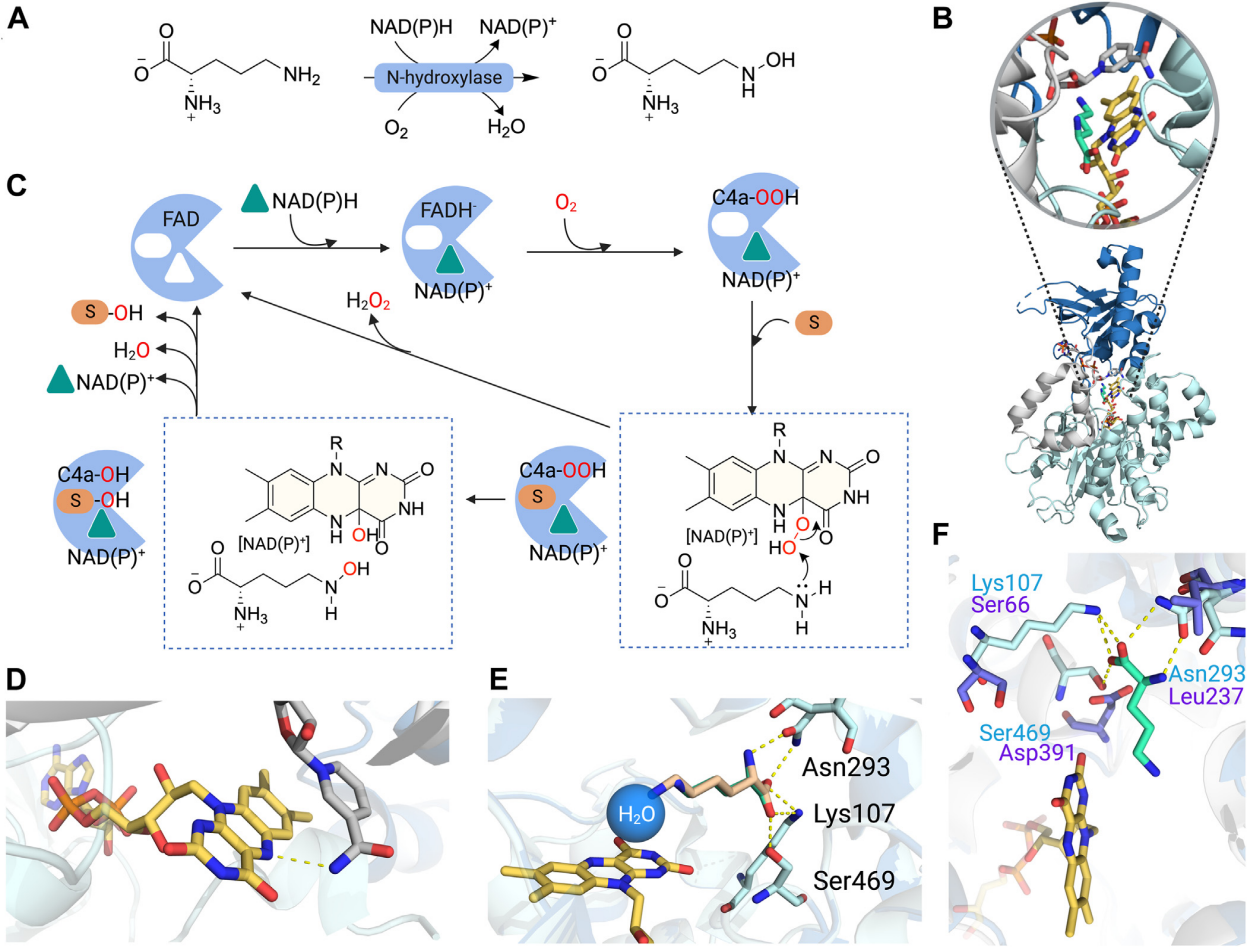
**Structures and reaction mechanisms of NMOs catalyzing N-hydroxylation.***A*, the reaction of N-hydroxylase catalyzing the hydroxylation of L-ornithine. *B*, the overall structure of SidA with FAD (*yellow sticks*), NADP^+^(*gray sticks*), and L-ornithine (*green sticks*) bound displaying a flavin-binding domain (*cyan*), NAD(P)^+^-binding domain (*blue*), and a substrate-binding domain (*gray*) (PDB: 4B63). *C*, the overall catalytic reaction of NMOs. *D*, the interaction of the N5-H isoalloxazine ring with the bound NADP^+^ in the SidA structure. *E*, key residues (*pale cyan sticks*) in the flavin- and substrate-binding sites of SidA. Overlaid structures of FAD, NADP^+^, and L-ornithine (*green sticks*) bound (PDB: 4B63) with FAD (*yellow sticks*), NADP^+^ (omitted in this figure) and L-lysine (*orange sticks*) bound SidA (PDB: 4B64). *F*, key residues in the flavin- and substrate-binding sites of *Ss*DesB (*purple sticks*) compared to SidA (*pale cyan sticks*). Overlaid structures of L-ornithine (*green sticks*)-bound SidA (PDB: 4B63) and *Ss*DesB with FAD (*yellow sticks*) and NADP^+^ (omitted in this figure) bound (PDB: 6XBB). FAD, flavin adenine dinucleotide; NMO, N-hydroxylating monooxygenase.

In addition to the ability to perform aromatic hydroxylation, FDMOs also exhibit the ability to hydroxylate various heteroatoms such as nitrogen, sulfur, and phosphorus (16). Among these, enzymes catalyzing the hydroxylation of nitrogen known as NMOs have recently been investigated extensively for their structural and mechanistic properties. NMOs belong to the single-component group B of FDMO (65, 66, 67, 68). The enzymes catalyze N-hydroxylation reactions of amine-containing compounds and use NAD(P)H as their reductant with a preference for NADPH over NADH (Fig. 4*A*) (65, 66, 67). NMOs also have the potential to be applied in biocatalysis applications for the production of N-O bond-containing compounds, which can subsequently be functionalized into N-N bonds. These chemical moieties are important in molecules exhibiting biological activities such as antifungal and antitumor activities (69). This section thus aims to highlight the key characteristics that enable NMOs to perform N-hydroxylation reactions. Specifically, we will focus on two extensively studied NMOs, L-ornithine hydroxylase from *A. fumigatus* (SidA) and L-ornithine hydroxylase from *Pseudomonas aeruginosa* (PvdA) (47, 70, 71, 72, 73).

The overall structure of NMOs comprises three distinct domains: the FAD-binding domain, the NAD(P)H-binding domain, and the substrate-binding domain. The active site is located at the interface between these three domains (Fig. 4*B*) (70, 71, 74). The catalytic process starts with the reduction of flavin by NAD(P)H, and unlike aromatic hydroxylases, NMOs do not require the prebinding of the substrate prior to flavin reduction (47, 72). Subsequently, the reduced enzyme, in complex with NAD(P)^+^, reacts with oxygen to generate a C4a-OOH as the reactive intermediate. This intermediate then transfers a terminal hydroxyl group onto a soft nucleophile amine-containing substrate, resulting in C4a-OH and a product with N-hydroxylation. The C4a-OH species subsequently decays to yield FAD and water. The water, NAD(P)^+^, and the N-hydroxylated product are then released from the active site, resetting the enzyme back to the oxidized FAD state ready for the next catalytic cycle (65). NMOs and other members of group B FDMOs are unique for their feature of having bound NAD(P)^+^ throughout the catalytic cycle (Fig. 4*C*) (75). The mechanistic advantage of this property is that the active site of NMOs appears to be exposed to outside solvent. The bound NAD(P)^+^ thus may help stabilize the C4a-OOH intermediate by providing H-bond interactions with the N5-H of C4a-OOH and by protecting the reactive C4a-OOH from reacting with outside solvent to eliminate H_2_O_2_ in the uncoupling pathway, ensuring the efficiency of the N-hydroxylation step (Fig. 4*D*) (71).

The efficiency of NMOs in catalyzing N-hydroxylation appears to primarily rely on the precise positioning of the substrate amine group that is undergoing hydroxylation and the C4a-OOH moiety. For instance, SidA demonstrates high specificity for L-ornithine and can also utilize L-lysine as a substrate. However, the *k*_cat_ of L-lysine hydroxylation is 8-fold lower than L-ornithine hydroxylation (70). Structural analyses of the SidA:NADP^+^:L-ornithine and SidA: NADP^+^:L-lysine complexes reveal that both L-ornithine and L-lysine adopt remarkably similar binding poses, with the exception of the terminal amine due to their differences in chain lengths (Fig. 4*E*) (70). The data imply that the binding position of L-ornithine is likely optimized for interacting with the distal oxygen of C4a-OOH as a water molecule observed in the SidA:NADP^+^:L-ornithine structure appears to mimic the position of the distal oxygen of C4a-OOH (Fig. 4*E*).

Site-directed mutagenesis experiments of SidA have identified several key active site residues crucial for efficient L-ornithine hydroxylation (76). The hydroxylation activity of the Lys107Ala variant is completely abolished, while the Asn293Ala and Ser469Ala variants exhibit weak substrate binding (Fig. 4*E*). The *k*_cat_ value for Asn293Ala was comparable, while the *k*_cat_ value for Ser469Ala was about 6-fold lower than that for the WT enzyme. The data imply that these residues are important for binding and orientation of the substrate in relation to the distal oxygen of C4a-OOH. Notably, recent research on cadaverine N-hydroxylase from *Streptomyces sviceus* (*Ss*DesB) catalyzing N-hydroxylation of a wide range of substrates has provided valuable insights (74). *Ss*DesB can hydroxylate alkyl diamines with methylene groups in the alkyl chain ranging from 4 to 7 units, that is cadaverine, putrescine, and spermidine. However, it displays limited activity towards L-lysine and does not hydroxylate L-ornithine. Structural analysis comparing SidA with *Ss*DesB reveals differences in the substrate-binding sites of these two enzymes (74). *Ss*DesB lacks residues with a positive charge or H-bond donor side chains that are supposed to anchor amino acid substrates in SidA such as Lys107, Asn293, and Ser469 (Fig. 4*F*). Additionally, *Ss*DesB contains Asp391 which can create charge repulsion with the carboxylate groups of amino acid substrates. Consequently, *Ss*DesB exhibits low hydroxylation activity towards amino acid substrates, underlining the critical role of substrate orientation with regards to C4a-OOH.

For future challenges, we think that more discovery of new NMOs with different substrate scopes may help in understanding the capability of their biocatalytic reactions. More enzyme engineering will also help explore the capabilities of these enzymes in catalyzing N-hydroxylation.

### Dehalogenation

In addition to a primary oxygenation reaction, certain FDMOs have been reported to exhibit an additional halogen atom or nitro group elimination activity. These FDMOs are generally referred to as flavin-dependent dehalogenases. Enzymes with these oxidative dehalogenation or denitration activities are members of group A (single-protein component type) and group D (two-protein component type) FDMOs (15, 16, 77). These enzymes are useful for bioremediation processes because they can catalyze the cleavage of carbon-halogen bonds in halogenated compounds, which are widely used as pesticides or in the chemical industry. Accumulation of these toxicants has posed serious environmental concerns because their biodegradability rate is slow without (bio)catalysts (78, 79).

**Figure 5:**
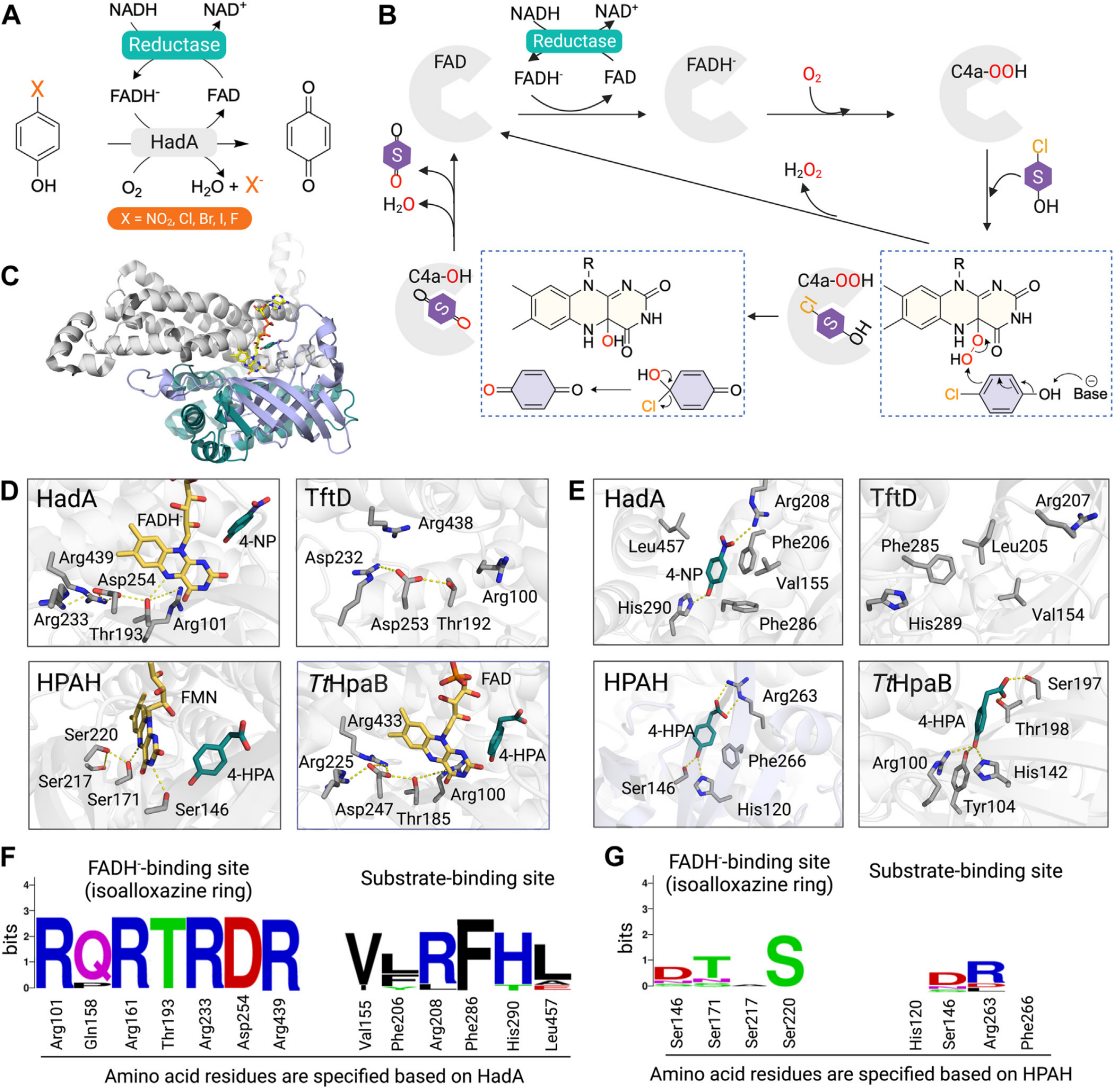
**Structures and reaction mechanisms of FDMO catalyzing dehalogenation.***A*, the reaction of flavin-dependent dehalogenase (HadA) catalyzing hydroxylation plus group removal from a phenolic derivative such as in the oxidative dehalogenation of 4-halophenol. *B*, the overall catalytic reaction of HadA. *C*, the overall structure of HadA (PDB: 7E8P). *D*, important residues at the flavin-binding site of two-component flavin-dependent dehalogenases (HadA from *Ralstonia pickettii* (PDB: 7E8P) and TftD from *Burkholderia cepacia* AC1100 (PDB: 3HWC)) and hydroxylases (HPAH from *A. baumannii* (PDB: 2JBT) and *Tt*HpaB from *Thermus thermophilus* HB8 (PDB: 2YYJ)). *E*, important residues at the substrate-binding site of two-component flavin-dependent dehalogenases (HadA and TftD) and hydroxylases (HPAH and *Tt*HpaB). *F*, conserved amino acid residues at a flavin-binding site and a substrate-binding site of two-component flavin-dependent dehalogenases. *G*, conserved amino acid residues at a flavin-binding site and a substrate-binding site of two-component flavin-dependent hydroxylases. The sequence analysis of *F* and *G* were analyzed using Clustal omega (EMBL-EBI) and the figures were created by WebLogo. The conserved residues in the flavin-binding site of dehalogenases and hydroxylases are similar, while the conserved residues in the substrate-binding site are different. FDMO, flavin-dependent monooxygenase.

Although several flavin-dependent dehalogenases have been reported for their abilities to catalyze oxidative dehalogenation, detailed information regarding their structural and mechanistic properties are not available (44, 80, 81, 82). Reaction kinetics and mechanistic studies have only been carried out for the reaction of HadA from *R. pickettii* DTP0602 (Fig. 5, *A* and *B*) (45, 83). Therefore, this section focuses on analyzing the mechanistic and structural data of this enzyme to gain insights into a dehalogenation reaction.

The overall reaction of dehalogenase consists of an initial step consisting of oxygenation or hydroxylation similar to the reaction of the hydroxylases described above and a second step consisting of a group elimination reaction. It is important to note that although they share common protein foldings, not all members of group A and D FDMOs can catalyze dehalogenation or group elimination reactions. Specific distinctions are required for catalyzing the additional group elimination reaction. Recently, structures of HadA with or without FADH^-^ and 4-nitrophenol (4-NP) bound have been reported and the reaction mechanisms of HadA reactions based on transient kinetics, substrate analogs, and site-directed mutagenesis are available (Fig. 5, *B* and *C*) (31). Therefore, analysis of HadA structures and mechanisms in comparison to those of PHBH and HPAH in the previous section (15, 16) should be informative for better understanding the catalytic features governing the additional group elimination reaction.

The overall kinetic mechanism of HadA involves formation of a C4a-OOH flavin intermediate similar to other FDMOs (Fig. 5*B*). The structure of HadA with FADH^-^ and 4-NP bound (Fig. 5*C*) (31) reveals the interactions between the flavin isoalloxazine ring and amino acid residues in the binding pocket including Arg101 and Thr193 through hydrogen bonding (Fig. 5*D*). Recently, site-directed mutagenesis results have also demonstrated that interactions of Arg101 with a neighboring residue, Asn447, are important for formation and stabilization of the C4a-OOH flavin (84). Thr193 forms a hydrogen bond with the N5-H of the flavin isoalloxazine ring and interacts with Asp254, which in turn forms a salt bridge with Arg233 and Arg439 (Fig. 5*D*). As previously mentioned, the hydrogen bonding to the N5-H atom of the isoalloxazine ring appears to be important for stabilization of C4a-(hydro)peroxyflavin in FDMOs. Site-directed mutagenesis results have also indicated that Thr193 and Asp254 play crucial roles in FADH^-^ binding, as well as in the formation and stabilization of C4a-OOH flavin. Alterations of these residues resulted in diminished activity of 4-NP conversion possibly due to the lower amount of C4a-OOH formed.

Herein, we performed sequences analysis of enzyme representatives of two-component FDMOs catalyzing hydroxylation plus dehalogenation and/or denitration including HadA from *R. pickettii* (45), dehalogenase from *Ralstonia eutropha* JMP134 (82, 85), 2,4,6-trichlorophenol monooxygenas from *Cupriavidus nantongensis* (86), TftD from *B. cepacia* AC1100 (44), 4-chlorophenol monooxygenase from *Arthrobacter chlorophenolicus* A6 (87), 4-nitrophenol 2-monooxygenase from *Rhodococcus opacus* (88), 4-nitrophenol monooxygenase from *Arthrobacter* sp. JS443 (89), 4-nitrophenol hydroxylase from *Rhodococcus* sp. PN1 (90), and 4-chlorophenol monooxygenase from *Rhodococcus* sp. JT-3 (91) in comparison to FDMOs catalyzing merely hydroxylation including HPAH from *A. baumannii* (30), *Tt*HpaB from *T. thermophilus* HB8 (43), *Ec*HpaB from *E. coli* (92), 4-hydroxyphenylacetate 3-hydroxylase from *P. aeruginosa* (39), 3-hydroxyphenylacetate 6-hydroxylase from *Pseudomonas putida* (93), 4-hydroxyphenylacetate hydroxylase from *Klebsiella pneumonia* (94), and phenol hydroxylase from *Bacillus thermoglucosidasius* A7 (95) to identify residues commonly found among the two types of enzymes and those distinctively found in the enzymes with dehalogenase or denitration activities. Previous results have shown that residues Arg101, Thr193, Arg233, Asp254, and Arg439 of HadA from *R. pickettii* (31) demonstrate homology with other FDMO members including TftD from *B. cepacia* AC1100 (44), as well as FDMOs catalyzing only hydroxylation (*e.g.*, *Tt*HpaB from *T. thermophilus* HB8 (43) and HPAH from *A. baumannii* (30)) (Fig. 5, *D*–*G*). These residues are closely located near the isoalloxazine-binding site, implying that residues surrounding the flavin site are not key determinants which differentiate between solely hydroxylation and hydroxylation plus additional group elimination.

The key determinant for controlling the group elimination in addition to the hydroxylation is likely located around the substrate-binding pocket which is a hydrophobic pocket. This pocket is comprised of hydrophobic residues including Val155, Phe206, Phe286, and Leu457 (Fig. 5*F*). Among these residues, Phe206 and Phe286 are particularly important, as substituting them with smaller hydrophobic amino acids (*i.e.* Val, Leu, Ile, or Ala) resulted in a significant decrease in 4-NP conversion activity (31). This suggests that the π-π interaction of Phe206 and the van der Waals contact of Phe286 with the substrate are crucial for the catalytic activity.

After conducting amino acid sequence and structure analysis, we observed notable distinctions between the two-component FDMO amino acid sequences and structures within the substrate-binding sites. Specifically, we found that residues that are highly conserved in the substrate-binding sites of flavin-dependent dehalogenases that exhibit hydroxylation, dehalogenation, and/or denitration activities are different from flavin-dependent monooxygenases that solely catalyze hydroxylation (Fig. 5, *E*–*G*). We hypothesize that the substrate-binding pocket plays a vital role in substrate accommodation and facilitating the proper geometric arrangement for enabling dehalogenation and denitration reactions in flavin-dependent dehalogenases. In addition to the residues involved in forming the hydrophobic pocket, His290 in HadA is also highly conserved among FDMOs with additional dehalogenation and denitration activities, but not in FDMOs that only catalyze hydroxylation. His290 is proposed to act as a catalytic base to abstract a proton from the hydroxyl group of the substrate, playing a crucial role in facilitating hydroxylation along with dehalogenation or denitration reactions in HadA (31). Although FDMOs such as *Tt*HpaB and HPAH, which solely catalyze hydroxylation, also contain a histidine residue near a hydroxyl group of the bound substrate, the position of this histidine residue differs from that of His290 in the HadA structure. It is worth noting that conformational changes may occur in HadA to facilitate the dehalogenation or group elimination reaction because the 4-NP–binding position based on the available structure is located beyond the reach of the -OH transfer from the C4a position of the isoalloxazine ring. This feature is different from the location of 4-HPA bound in HPAH (Fig. 5*E*) (30, 31).

Collectively, the residues within the substrate-binding pocket are key factors dictating substrate accommodation and facilitating the proper geometric arrangement which enables dehalogenation and denitration reactions in FDMOs. It would be interesting to explore the possibility of converting flavin-dependent hydroxylases into dehalogenases by engineering the substrate-binding site in the future.

### Halogenation

**Figure 6:**
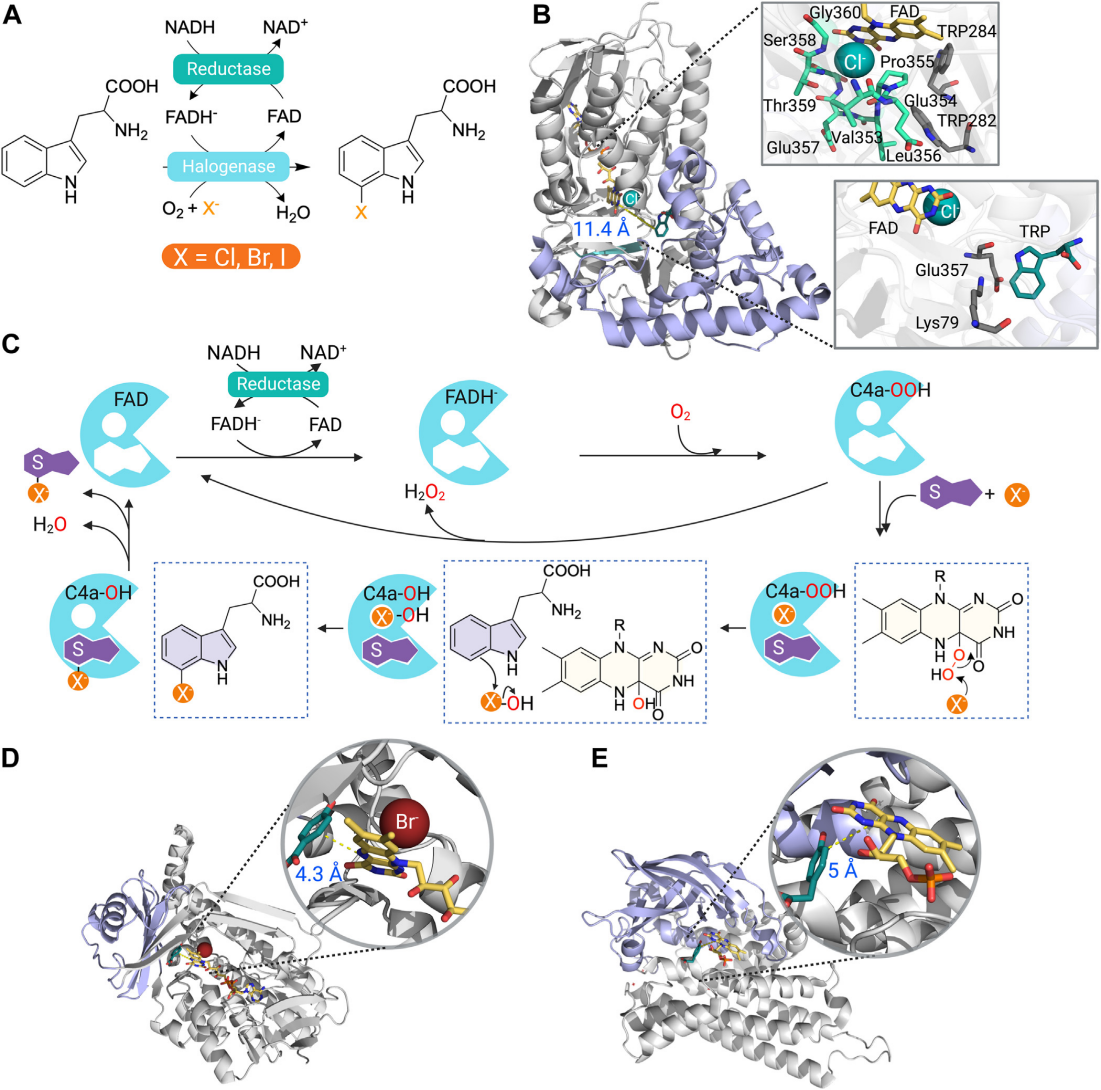
**Structure and reaction mechanisms of FDMO catalyzing halogenation.***A*, the reaction of halogenase catalyzing halogenation of tryptophan. *B*, the structure of RebH with FAD (*yellow sticks*), chloride (*green sphere*), and tryptophan bound (*green sticks*) (PDB: 2OA1). The structure comprises of the flavin-binding (*gray*) and substrate-binding domains (*purple*). The conserved region, WxWxIP, is colored as a *green* β-sheet strand. Key amino acid residues at the substrate-binding site and flavin-binding site are represented as *gray sticks*. Amino acid residues at chloride-binding site are represented as *green sticks*. *C*, the catalytic cycle of flavin-dependent halogenase. *D*, the structure of single-component hydroxylase, PHBH, with FAD (*yellow sticks*), 4-HBA (*green sticks*), and bromide bound (*red sphere*) (PDB: 1DOE). *E*, the structure of two-component hydroxylase, HPAH, with FMN (*yellow sticks*) and 4-HPA (*green sticks*) bound (PDB: 2JBT). FAD, flavin adenine dinucleotide; FDMO, flavin-dependent monooxygenase; FMN, flavin mononucleotide.

Flavin-dependent halogenase belongs to group F of the FDMO family which contains the majority of flavin-dependent halogenases that are two-component enzymes (16, 20, 56, 96). The enzymes catalyze the regioselective halogenation of a wide range of aromatic and aliphatic substrates using halide ions and molecular oxygen (Fig. 6*A*) (97, 98, 99). Flavin-dependent halogenases are noteworthy exceptions within the FDMOs in that they generate halogenated instead of oxygenated products (16).

Tryptophan halogenases catalyzing the halogenation of tryptophan are the enzymes which have undergone the most comprehensive investigation within the family (96). Transient kinetic studies of tryptophan 7-halogenase (RebH) and tryptophan 6-halogenase (Thal) reveal that the first step of the reactions is the enzyme-bound FADH^-^ reacting with oxygen to form a C4a-OOH flavin intermediate (24, 100). This intermediate can then react with halide ions (Cl^-^, Br^-^, or I^-^) to form hypohalous acid (HOX) and a C4a-OH flavin intermediate (24). Water elimination from the C4a-OH flavin intermediate then occurs to yield an oxidized flavin. The presence of the tryptophan substrate has no impact on flavin reactions (24, 100, 101), confirming that tryptophan does not directly participate in the hydroxylation of substrate by the C4a-OOH flavin intermediate. In fact, tryptophan binds at the different active site. These properties are key distinctions which differentiate the flavin-dependent halogenases from hydroxylases, contributing to the underlying mechanisms enabling halogenation instead of hydroxylation (102).

During the past decade, several crystal structures of flavin-dependent halogenases have been reported (96, 103). All flavin-dependent halogenases consist of two separated binding sites which are different from the other FDMO members (Fig. 6*B*). All enzymes share a common flavin-binding architecture called a Rossman fold (Fig. 6*B*) (96, 98), while they lie within the substrate-binding site which displays variation in length and folding. This structural variability allows flavin-dependent halogenases to utilize a wide array of substrates including indole, pyrrole, and phenolic substrates (96, 103). The structures of tryptophan halogenases reveal that the FAD- and tryptophan-binding sites are separated by about 10 Å (Fig. 6*B*) (104, 105, 106, 107). The halide ion was found on the *re*-side of the flavin close to the C4a position.

Based on the combined analysis of structural and transient kinetics data, it was proposed that C4a-OOH flavin reacts with a halide ion located in close proximity to the flavin, resulting in the formation of HOX (Fig. 6, *B* and *C*) (24, 100, 101, 104). Subsequently, HOX diffuses through a tunnel and reaches the second active site to interact with a strictly conserved catalytic residue, lysine, which is approximately 10 Å away. The catalytic lysine is crucial for aromatic substrate halogenation, and replacing this residue with other amino acids leads to an inactive enzyme for halogenation even though the C4a-OOH flavin can be formed (24). It was previously proposed that the conserved lysine reacts with HOX to form a long-live chloramine intermediate (-NH_2_Cl^+^ or -NHCl) (35). However, subsequent evidence indicates that the catalytic lysine is fully protonated, establishing a hydrogen bond and serving as a proton donor to HOX during catalysis (24, 108, 109). The model is supported by the unusually low p*K*a value obtained from calculations of the catalytic lysine compared to other lysines in the structure (24). This mechanistic explanation aligns with a model proposed based on quantum mechanical/molecular mechanical and density functional theory (DFT), which observed a hydrogen bond forming between a hydrogen of the catalytic lysine and the oxygen of HOX (108). Recent investigations into the functional role of the catalytic lysine using molecular dynamics simulations and DFT calculations also support the importance of a protonated lysine for stable HOX binding (109). Additionally, DFT calculations reveal that the energy barrier for the formation of the chloramine intermediate is significantly higher than the energy required for hydrogen bond formation between the catalytic lysine and HOX (109). Taken together, these findings suggest that the catalytic lysine most likely interacts with HOX *via* hydrogen bonding to facilitate halogenation.

Flavin-dependent halogenases have two strongly conserved stretches of amino acids within the flavin-binding site (GxGxxG and WxWxIP) and at the tryptophan-binding site consisting of a WxWxIP motif. We propose that the presence of the WxWxIP motif in two-component halogenases is a key structural feature which prevents the enzymes from carrying out hydroxylation reactions and facilitates halogenation reactions. The conserved WxWxIP was proposed to provide a shield for the tryptophan-binding site, thus preventing aromatic hydroxylation from occurring in the flavin-dependent halogenase (96, 103). For single-component hydroxylases such as PHBH or two-component hydroxylases such as HPAH, aromatic substrates bind closely to the flavin C4a-position at distances of 4.3 Å and 5 Å, respectively (Fig. 6, *D* and *E*). It should be noted that although halide ion binding is found near the isoalloxazine ring of the flavin molecule in the crystal structure of PHBH from *P*. *aeruginosa* (Fig. 6*D*) (110), the enzyme could not carry out halogenation. This is possibly due to the presence of an aromatic compound (which is required for flavin reduction stimulation (Fig. 6*D*) in the active site prior to the formation of the C4a-OOH flavin intermediate. Once the intermediate is formed, the hydroxyl group transfer from the C4a-OOH flavin to the bound substrate can readily take place. It is also possible that specific interactions between binding residues and substrate may be required to facilitate halogenation. For flavin-dependent halogenases, although the WxWxIP motif has been proposed to separate the two active sites, the specific function of certain residues within this motif towards halogenation remains unclear.

For future challenges, we think that further exploring the engineering of the WxWxIP motif may provide a more complete understanding of the halogenation mechanisms and identify the requirements of binding residues to support halogenation. It may also be interesting to exploring changes in the substrate-binding environment which may convert a halogenase into a hydroxylase or vice versa.

### Light emission

**Figure 7:**
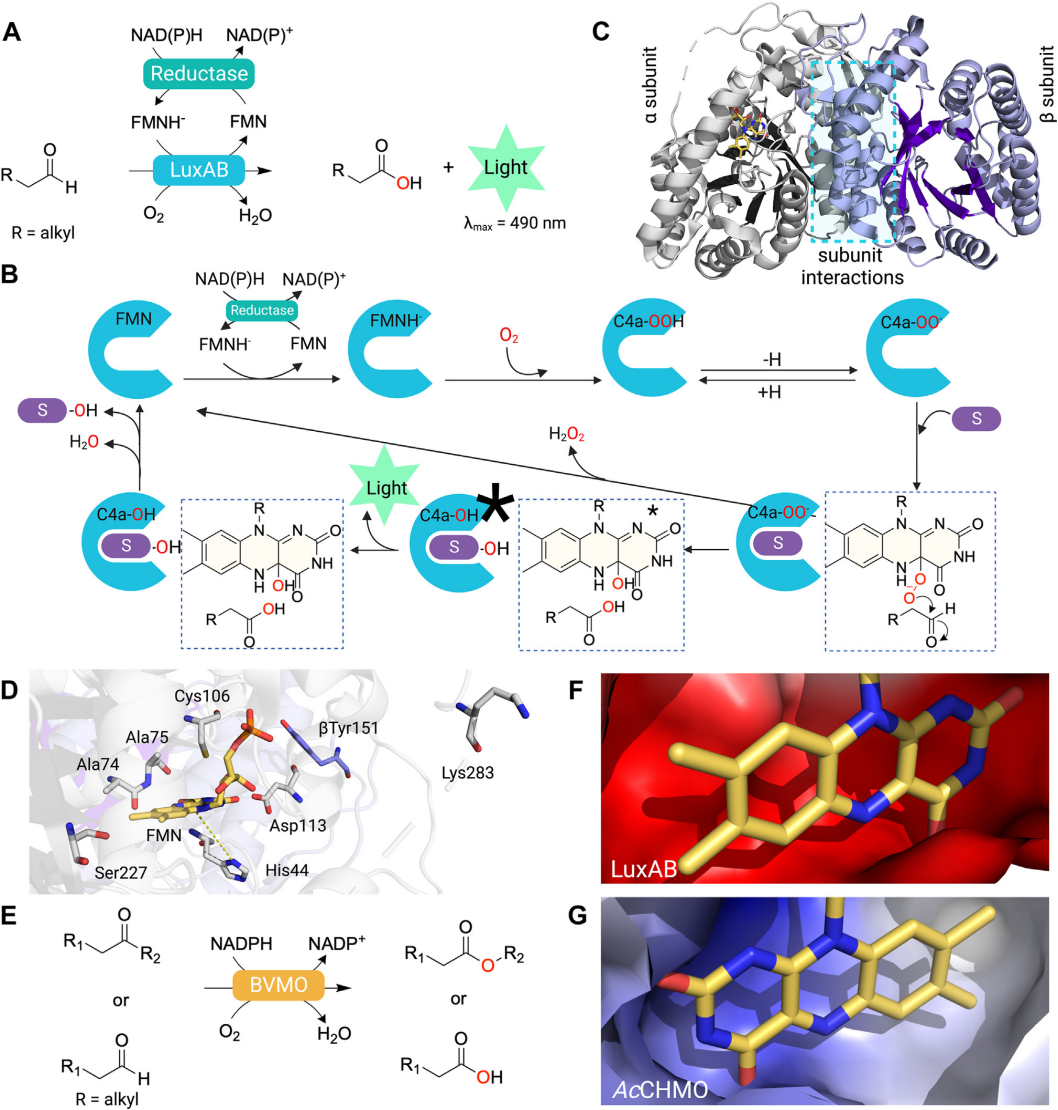
**Structures and reaction mechanisms of FDMO catalyzing hydroxylation and light emission.***A*, the reaction of LuxAB catalyzing light emission and oxidation of long-chain aldehydes. *B*, the reaction mechanism of LuxAB. *C*, the α-subunit (*gray*) and β-subunit (*purple*) which comprise the structure of LuxAB (PDB: 3FGC). Both subunits contain a TIM barrel folding structure (*dark gray* and *dark purple*, respectively). *D*, keys residues at the active site of LuxAB. *E*, the reaction of BVMO catalyzing oxidation of ketones or aldehydes. *F*, electrostatic potentials in the active site of LuxAB (PDB: 3FGC). *G*, electrostatic potential in the active site of cyclohexanone monooxygenase from *Acinetobacter calcoaceticus* (*Ac*CHMO) (PDB: 6A37) calculated using APBS calculation at pH7.0 (https://www.poissonboltzmann.org/). BVMO, Baeyer–Villiger monooxygenase; CHMO, cyclohexanone monooxygenase.

Among the enzymes in the FDMO family, bacterial luciferase (LuxAB) stands out for its ability to catalyze oxygenation and light emission simultaneously. This enzyme catalyzes the bioconversion of long-chain aldehyde and molecular oxygen to carboxylic acid and water, using reduced FMN (FMNH^−^) as a substrate (111, 112, 113). Along with carboxylic acid and water, bacterial luciferase emits light with a maximum emission (λ_max_) of around 490 nm (16, 111, 114). The overall reaction of bacterial luciferase is shown in Figure 7, *A* and *B*. Briefly, bacterial luciferase initiates the catalytic cycle by generating C4a-OOH flavin, which is subsequently deprotonated to generate C4a-peroxyflavin (C4a-OO^-^). This C4a-OO^-^ flavin then reacts with an aldehyde substrate, resulting in the formation of a C4a-peroxyhemiacetal flavin. This compound undergoes O − O bond cleavage, giving rise to the excited state C4a-hydroxyflavin (C4a-OH∗), which emits light as a luminescent species (Fig. 7*B*). This section highlights the distinct features of the structures and mechanisms of bacterial luciferase. Data from site-directed mutagenesis studies of Lux from *Vibrio harveyi* or *Vibrio campbellii* are instrumental to better understanding the structure and function relationship of bacterial luciferase.

LuxAB can be classified as a two-component FDMO because it requires a flavin reductase to generate FMNH^-^ as a substrate for its oxygenation reaction. Although the native flavin reductase is encoded in the same operon as LuxAB is LuxG, other flavin reductases can also serve to supply FMNH^-^ to the enzyme, as no specific protein interactions are required for the flavin transfer process (115, 116, 117). LuxAB consists of α and β subunits that have the same TIM barrel folds (Fig. 7*C*). The catalytic activity is carried out mainly by the α subunit, while the noncatalytic β subunit stabilizes the catalytic subunit *via* the interaction of the α2 and α3 helices of each subunit (118). The FMN-bound LuxAB structure together with site-directed mutagenesis have identified important functions of the residues around the FMNH^-^-binding pocket (118). Mutation of residues including αHis44, αAla74, αAla75, αCys106, αAsp113, αSer227, αLys283, αLys286, and βTyr151 affected the stability of the C4a-OO(H) flavin intermediate and resulted in the reduction of bioluminescence activity (Fig. 7*D*) (111). Mutation of the conserved αHis44 in LuxAB from *V. harveyi* located near the isoalloxazine ring to Ala resulted in a reduction of light production by about six orders of magnitude (119). Recently, investigation of LuxAB from *V. campbellii* using transient kinetics shows that the αHis44Ala, αHis44Asp, and αHis44Asn variants can form the C4a-OOH flavin but the intermediate could not proceed to generate bioluminescence (120). The recent study using quantum mechanical/molecular mechanical calculations revealed that the conserved His44 is crucial for bioluminescence activity because it acts as a proton acceptor from the C4a-OOH flavin to promote formation of the reactive flavin intermediate C4a-OO^-^ flavin. The C4a-OO^-^ flavin serves as a nucleophile to attack an aldehyde substrate, leading to the formation of a C4a-peroxyhemiacetal flavin (Fig. 7*B*) (121, 122). This intermediate undergoes O − O bond cleavage, producing carboxylic acid and the excited state C4a-hydroxyflavin (C4a-OH∗) as an active luminescent species which emits light by transferring the excited state electron of C4a-OH flavin to the ground state level (123). The C4a-OH flavin then eliminates water to form oxidized flavin as a final product (Fig. 7*B*). In the absence of aldehyde, the reaction proceeds through an uncoupled or dark pathway by eliminating H_2_O_2_ to generate oxidized flavin as in the uncoupled pathway of FDMOs previously discussed (Figs. 3, *E* and *F*, 4*C*, 5*B*, 6*C*, and 7*B*). It is intriguing to note that the ability to promote formation of the excited state C4a-hydroxyflavin is only found in the active site architecture of LuxAB and not in other FDMOs.

The reaction mechanism underlying the generation of the excited light-emitting species (C4a-OH∗) is among the most challenging questions of the field and the mechanism is still under debate. Several mechanisms to explain this reaction have been proposed including Baeyer–Villiger rearrangement, dissociative electron transfer, chemically initiated electron exchange luminescence, charge-transfer initiated luminescence (CTIL), dioxirane, upper excited state formation, and peroxy ester formation (111, 124). Recent studies utilizing DFT calculations to investigate energetics during C4a-OH∗ flavin formation in all seven proposed mechanisms suggested that the formation of C4a-OH∗ flavin likely proceeds *via* a CTIL mechanism (124).

The CTIL mechanism proposes that the C4a-OH∗ flavin undergoes relaxation to the ground state *via* conical intersection (CI), a point where the energy of the same molecule in different electronic states, such as the ground state and an excited state, intersect. During this relaxation process from the excited state (C4a-OH∗), charge transfer (electron delocalization) to the pyrimidine ring moiety occurs, leading to a dissipation in energy without photon emission. Consequently, this charge transfer mechanism effectively suppresses light emission. The study using minimum-energy path calculations of the excited state C4a-OH∗ flavin relaxation in a liquid solvent and frozen solvent found that C4a-OH∗ flavin in the frozen solvent restrains ring distortion and charge transfer to the pyrimidine ring moiety, thus blocking the CI-mediated internal conversion, which resulted in a high fluorescence quantum yield (125). Therefore, it was proposed that this CI is not observed in the protein, preventing the energy reduction without photon emission and in turn promotes the high luminescence quantum yield observed in bacterial luciferase (126).

Interestingly, although LuxAB and BVMOs both use the same C4a-OO^-^ flavin to catalyze nucleophilic attack of an electrophile substrate, BVMOs only catalyze oxygenation without light emission (7, 127, 128). BVMOs catalyze an oxygen insertion of diverse substrates such as aldehydes, ketones, amines, and organoboranes (127, 129). Products of BVMO reactions are oxygenated compounds (*i.e.*, esters, lactones, acids depending on substrates) and water, similar to those in the luciferase reaction (Fig. 7, *A* and *E*) (127, 129).

We thus compared the protein environment around the isoalloxazine ring of bacterial luciferase and in cyclohexanone monooxygenase from *Acinetobacter calcoaceticus* (*Ac*CHMO) using the electrostatic potential calculations. Briefly, crystal structure of LuxAB (PDB: 3FGC) and *Ac*CHMO (PDB: 6A37) were prepared by the PDB2PQR software (https://server.poissonboltzmann.org/pdb2pqr) to add missing atoms and assign charges and radii at pH 7.0 (130). The output structures from PDB2PQR were then further subjected to the APBS (Adaptive Poisson-Boltzmann Solver) software (https://server.poissonboltzmann.org/apbs) for electrostatic potential calculations (131). The data revealed that the isoalloxazine pocket of bacterial luciferase has a negative electrostatic potential (Fig. 7*F*) while *Ac*CHMO does not (Fig. 7*G*). The negative electrostatic potential found in LuxAB may destabilize the charge transfer process (electron delocalization) to the pyrimidine ring moiety which leads to the CI of excited state C4a-OH∗ flavin relaxation where the energy of the ground state and an excited state is degeneracy and promotes energy reduction without light emission; this promotes a luminescence phenomenon instead. On the other hand, the isoalloxazine ring pocket of the *Ac*CHMO has a positive electrostatic potential, thus stabilizing the charge transfer process and promoting the CI process, resulting in a decrease in the luminescence quantum yield. Overall, this analysis provides explanation why luminescence is not observed in the BVMO enzymes.

In our opinion, enzyme engineering approaches would be interesting to employ for future investigation whether the activity of another enzyme within the FDMO family can be adjusted to produce bioluminescence. It is worth mentioning that the mechanisms underlying the light-emitting reaction remained elusive. The DFT calculations were only conducted to mimic solvent environment and not in the actual enzyme active site (124). It would be premature to dismiss all of the previously proposed mechanisms. Additional investigations are still required to gather compelling evidence and establish the reaction mechanisms conclusively.

## Conclusion and perspectives

Flavin-dependent monooxygenases are highly complex enzymes that play crucial roles in nature and serve as remarkable biocatalysts with broad applications. Their green chemistry, selectivity, robustness, and versatility make them valuable biocatalysts in various fields (1, 3, 20). Although FDMOs exhibit remarkable structural diversity, they share common recognition features for the flavin cofactor and a well-defined environment surrounding the C4a-N5 locus (Fig. 2*N*). This environment includes a substrate-binding pocket and molecular oxygen–binding pocket above the C4a-flavin and residues capable of forming hydrogen bonds with the N5-flavin. These elements facilitate the formation of a common reactive C4a-(hydro)peroxyflavin species, which plays a crucial role in substrate functionalization (1, 2).

**Figure 8:**
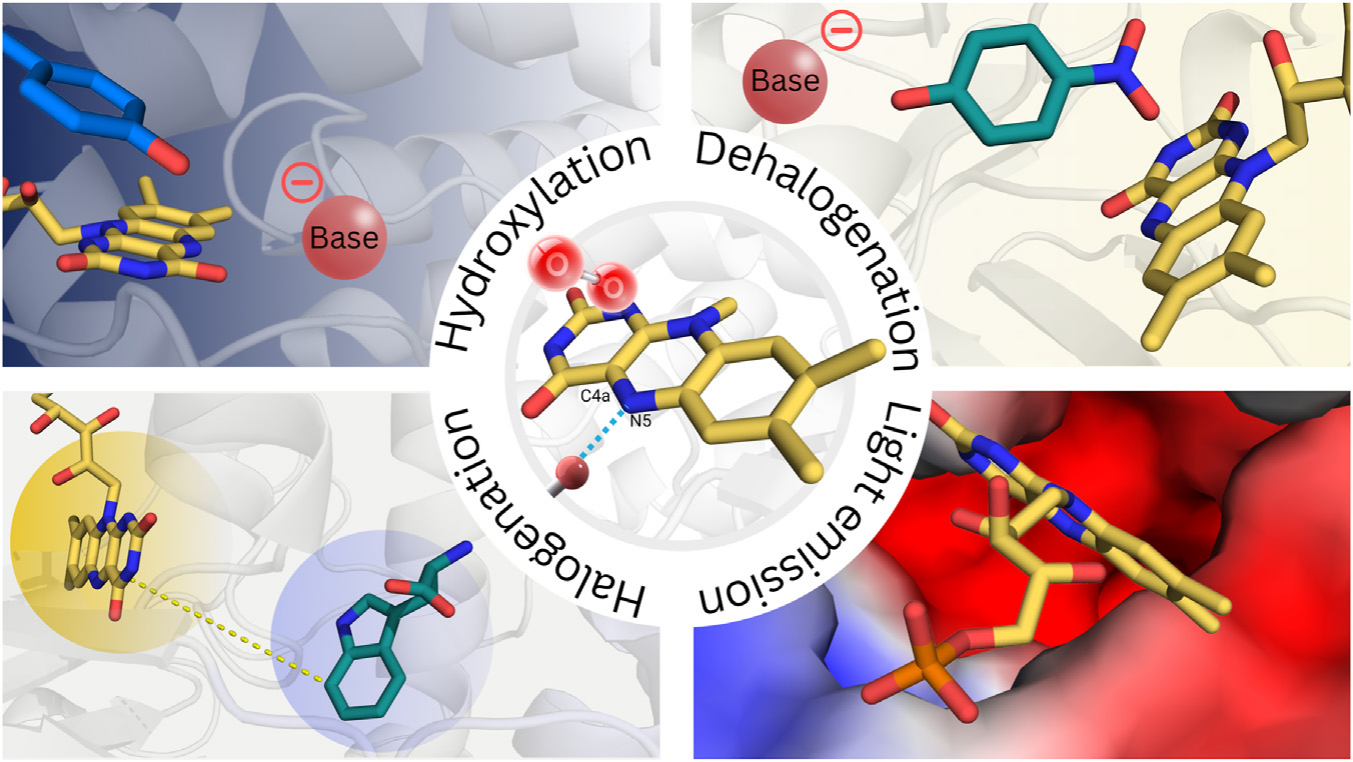
**A comprehensive overview of the essential attributes of FDMOs in handling the versatility of the C4a-(hydro)proxyflavin intermediate, enabling catalysis of a diverse spectrum of reactions.** FDMO, flavin-dependent monooxygenase.

Extensive research on FDMOs has uncovered distinctive features that potentially govern the versatility of C4a-(hydro)peroxyflavin in diverse reactions, such as hydroxylation, dehalogenation, halogenation, and light emission. The architecture of the substrate-binding site appears to be a critical determinant for the hydroxylation (both aromatic hydroxylation and N-hydroxylation) or dehalogenation activity in flavin-dependent aromatic hydroxylases, N-hydroxylating flavin-dependent monooxygenases, and flavin-dependent dehalogenases, respectively. The presence of a substrate-binding pocket that facilitates substrate deprotonation at the hydroxyl group is crucial for efficient hydroxylation in both flavin-dependent aromatic hydroxylases and flavin-dependent dehalogenases (Fig. 8). For N-hydroxylating hydroxylases, active site residues to facilitate substrate deprotonation are not strictly required. This is due to the intrinsic soft nucleophilic property of the amine nitrogen. In these enzymes, the substrate pocket that governs the precise positioning of the substrate amine group towards the C4a-hydroperoxyflavin is vital for their hydroxylation activity. Nevertheless, in the case of flavin-dependent dehalogenases, which exhibit an additional group elimination activity beyond hydroxylation, the amino acid residues constituting the hydrophobic substrate-binding pocket play a pivotal role in accommodating the substrate and enabling the precise geometric configuration required for effective dehalogenation (Fig. 8). In the case of flavin-dependent halogenases, the separation of the flavin-binding site and the substrate-binding site seems to play a pivotal role in halogenation activity (Fig. 8). For bacterial luciferase, the electrostatic potential of the flavin-binding site is proposed to be a significant factor influencing light emission activity (Fig. 8).

Despite significant progress in understanding FDMOs, current knowledge has not yet enabled the rational redesign of FDMOs to switch their activities. This challenge stems from the inherent complexity of FDMOs, and successful instances of activity switching in FDMOs have not been reported to date. Future challenges in the field involve further investigation of these complexities and taking the necessary steps towards enabling efficient enzyme redesign for biotechnological applications.

## Conflict of interest

The authors declare that they have no conflicts of interest with the contents of this article.
